# 1,25-Dihydroxyvitamin D3 prevents bone loss of the secondary spongiosa in arthritic rats by an increase of bone formation and mineralization and inhibition of bone resorption

**DOI:** 10.1186/1471-2474-15-345

**Published:** 2014-10-14

**Authors:** Peter Oelzner, Peter K Petrow, Gunter Wolf, Rolf Bräuer

**Affiliations:** Department of Internal Medicine III, University Hospital of Jena, Erlanger Allee 101, 07740 Jena, Germany; Institute of Pathology, University Hospital of Jena, Ziegelmühlenweg 1, 07740 Jena, Germany

**Keywords:** Arthritis models, Bone, Vitamin D hormone

## Abstract

**Background:**

Active vitamin D metabolites have been shown to have protective effects in experimental arthritis especially when used as preventive treatment. However, because the direct effects of 1,25-dihydroxyvitamin D3 (1,25(OH) _2_D_3_) on bone formation and resorption are very complex, the net effect of 1,25(OH)_2_D_3_ on histomorphometric parameters of bone turnover and mineralisation should be investigated. Therefore, we examined the influence of 1,25(OH)_2_D_3_ therapy on arthritis-induced alterations of periarticular and axial bone as well as disease activity, inflammation and joint destruction in antigen-induced arthritis (AIA) of the rat.

**Methods:**

AIA was induced in 20 eight-week-old female Wistar rats. 10 rats without arthritis were used as healthy controls. AIA rats received 1,25(OH)_2_D_3_ (0.2 μg/kg/day, i.p., n = 10) or vehicle (n = 10) at regular intervals for 28 consecutive days beginning 3 days before arthritis induction. Bone structure of the secondary spongiosa of the periarticular and axial bone was analyzed using histomorphometry. Parameters of mineralization were investigated using tetracycline labelling. Clinical disease activity, inflammation and joint destruction were measured by joint swelling and histological investigation, respectively.

**Results:**

AIA led to significant periarticular bone loss. 1,25(OH)_2_D_3_ treatment resulted in a highly significant increase in trabecular bone volume and bone formation rate in comparison to both vehicle-treated AIA and healthy controls at periarticular (p < 0.01 and p < 0.001, respectively) and axial bone (p < 0.001 and p < 0.001, respectively). In addition, bone resorption was reduced by 1,25(OH)_2_D_3_ at the axial bone (p < 0.05 vs. vehicle-treated AIA). Joint swelling as well as histological signs of inflammation and joint destruction were not influenced by 1,25(OH)_2_D_3_.

**Conclusions:**

The results of the study indicate a marked osteoanabolic effect of 1,25(OH)_2_D_3_ presumably due to a substantial increase in mineralization. Thus, 1,25(OH)_2_D_3_ may be an effective osteoanabolic treatment principle to antagonize the inflammation-associated suppression of bone formation in rheumatoid arthritis.

**Electronic supplementary material:**

The online version of this article (doi:10.1186/1471-2474-15-345) contains supplementary material, which is available to authorized users.

## Background

Rheumatoid arthritis (RA) is characterized by early periarticular demineralization followed by periarticular bone destruction and often complicated by systemic bone loss resulting in fractures [1–3]. Both increased osteoclastic bone resorption due to an imbalance between the production of receptor activator of NFκB ligand (RANKL) which stimulates bone resorption and its decoy receptor osteoprotegerin (OPG) [4–8] and decreased bone formation and repair mediated by increased production of wnt inhibitors [8–11] contribute to bone destruction and systemic osteoporosis. Furthermore, the finding that the knockout of nuclear factor erythroid 2-related factor 2 (Nrf2), a transcription factor that maintains the cellular defence against oxidative stress, in mice with antibody-induced arthritis was associated not only with an increase in cartilage destruction but also with a high number of spontaneous fractures underlines the importance of reactive oxygen species for bone damage in arthritis models [12]. Based on these pathogenetic mechanisms, which are often only incompletely suppressed by immunosuppressive therapy, adjuvant treatment of RA with substances, that are potentially able to prevent bone loss, is of particular interest. The vitamin D hormone 1,25-dihydroxycholecalciferol (1,25(OH)_2_D_3_) has been shown to induce osteoblast differentiation [13–15]. Furthermore, 1,25(OH)_2_D_3_ acts as an antiinflammatory substance by marked influences on T cell differentiation with a suppression of Th1 and Th17 cells and induction of differentiation into Th2 und regulatory T cells as well as by suppression of costimulation receptors on antigen-presenting cells, inhibition of differentiation of dendritic cells and inhibition of NFκB activation, p38 activation and cytokine production in monocytes/macrophages and suppression of angiogenesis [16–23]. RA is often associated with vitamin D deficiency and serum levels of 1,25(OH)_2_D_3_ have been shown to decrease in patients with high disease activity [24, 25]. Therefore, 1,25(OH)_2_D_3_ may have a beneficial effect on both bone metabolism and inflammation in RA and animal models of this disease. A very detailed and comprehensive analysis regarding immunological processes, cytokines involved in bone resorption and bone turnover was performed in vitamin D receptor (VDR) knockout mice with spontaneously developing arthritis [20]. Furthermore, a preventive or protective effect of the treatment with 1,25(OH)_2_D_3_ or with other active vitamin D metabolites in experimental arthritis (animal models of RA) has been shown [26–34]. However, most of the cited studies focused on incidence, severity and duration of arthritis [29–33] including detailed investigation of immunological mechanisms of arthritis [34] and an analysis of bone was not performed or bone turnover was not measured using histomorphometry. A detailed investigation of the effect of 1,25(OH)_2_D_3_ on bone turnover is necessary because the direct effects of 1,25(OH)_2_D_3_ on bone include the expression of RANKL which may result in increased bone resorption [35], whereas bone resorption could be suppressed indirectly by inhibition of inflammation [20]. To determine the net effect of 1,25(OH)_2_D_3_ on bone and inflammation, we investigated the influence of 1,25(OH)_2_D_3_ on histomorphometric parameters of bone turnover and mineralisation at periarticular and axial bone as well as on inflammatory disease activity in antigen-induced arthritis (AIA) of the rat, a T cell-dependent model of RA [36, 37].

## Methods

### Arthritis induction

Eight-week-old female Wistar rats (Central Animal Research Facility, University Hospital, Jena, Germany) maintained under standardized conditions were subjected to a 12 h/12 h light/darkness cycle and fed with pellet food (Altromin, No 1326, Lage, Germany) and water ad libitum.

Because of the complex regulation of vitamin D metabolism including both a strongly regulated renal synthesis of 1,25(OH)_2_D_3_ and a substrate dependent 1,25(OH)_2_D_3_ synthesis in osteoblasts and immune cells [13–15, 19], 1,25(OH)_2_D_3_ effects could also be influenced by vitamin D intake. To keep the influence of vitamin D intake on the results of our experiment constant, a diet containing a physiological and standardized concentration of vitamin D was started in arthritic animals before arthritis induction and also in healthy animals.

The diet contained 0.9% calcium (0.9 g calcium/100 g), 0.7% phosphorus (0.7 g phosphorus/100 g) and 600 IU vitamin D3 per kg. With respect to vitamin D3 and calcium content this is a conventional diet comparable to those used by Vieth et al. [38].

The animals were subcutaneously immunized with 0.5 mg of methylated bovine serum albumin (mBSA, Sigma, Deisenhofen, Germany) in 0.5 ml of saline and emulsified in 0.5 ml of complete Freund’s adjuvant (Sigma), containing 2 mg/ml of heat-killed Mycobacterium tuberculosis strain H37RA (Difco, Detroit, MI, USA) 21 and 14 days before AIA induction.

Arthritis was elicited by injecting 0.5 mg mBSA in 50 μl sterile phosphate-buffered saline (PBS) into the right knee joint cavity. The same volume of PBS was injected into the left knee as an intra-individual control. Ethical guidelines for experimental investigations in animals were used [39]. All procedures complied with the regulations of the Thuringian Commission for Animal Protection. The approval of our local ethics committee was obtained for our study.

### Drug administration

Rats with AIA were divided into 2 groups to receive intraperitoneal injections before and after AIA induction according to the following regimens:

Group 1: 1,25(OH)_2_D_3_ (Calcijex, Abbott, Chicago USA), 0.2 μg/kg beginning three days before arthritis induction up to day 15 after arthritis induction every day and after day 15 every other day. The cumulative dose of 1,25(OH)_2_D_3_ in this group was 6.5 μg/kg.

Group 2: 50 μl of solvent for 1,25(OH)_2_D_3_ (vehicle) beginning three days before arthritis induction up to day 15 after arthritis induction every day and after day 15 every other day (untreated AIA; n = 10). The solvent contains 20.4 mg/ml polysorbate and 2.5 mg/ml sodium ascorbate.

The time point to start 1,25(OH)_2_D_3_ application in our experiment three days before AIA induction was determined by both using more a therapeutic than a prophylactic regimen of 1,25(OH)_2_D_3_ administration and to achieve a complete treatment effect in the early acute phase of AIA. At this time point the immunologic changes characteristic for AIA were established [36, 37]. On the other hand, regarding the time course of AIA with an early acute phase with high disease activity and a longer chronic phase with lower disease activity we targeted a complete treatment effect in the early acute phase of the arthritis.

Additionally, ten healthy animals (without immunization and AIA) were used as healthy controls.

The serum levels of vitamin D were not measured throughout the experiment.

### Assessment of arthritis

Arthritis was monitored by measuring the mediolateral joint diameter using a vernier caliper [40]. Swelling was expressed as the difference in mm between the right arthritic and the left reference joint at the days 3, 7, 14, 22 and 28 after arthritis induction.

### Preparation of bones for histomorphometric and histopathologic analysis

All 20 arthritic rats, as well as 10 healthy age-matched controls, were sacrificed on day 28 after AIA induction. Both the right tibia head of the arthritic knee joint and the third lumbar vertebra were removed and used for histomorphometric analysis. After preparation, the bones were fixed in acetone for 24 hours and embedded using the embedding system Technovit 9100 NEU (Heraeus-Kulzer, Wehrheim, Germany) for mineralized tissue. In principal, the system is based on chemical polymerization employing a catalytic system consisting of peroxide and amine without oxygen. A Polycut S-Special Microtome was used to cut 5 μm thick sections (Jung/Leica, Heidelberg, Germany). Trichrome Masson/Goldner staining was performed to differentiate mineralized bone and osteoid [41]. In addition, Giemsa staining was performed to allow distinction of the cellular components of bone tissue.

### Histopathologic assessment of inflammation and joint destruction

Cross sections from knee joints were stained with hematoxylin and eosin (HE) and were evaluated using a photomicroscope (Axioskop 2, Carl Zeiss, Jena, Germany). To analyze the inflammatory and destructive activity of arthritis, knee joint sections were examined in a blind fashion using a semiquantitative score (0 = no, 1 = mild, 2 = moderate, 3 = severe alterations) for the extent of acute (quantity of fibrin exudation and relative number and density of granulocytes in the synovial membrane and in joint space) and chronic inflammatory changes (relative number and density of infiltrating mononuclear leukocytes in the synovial membrane, degree of synovial hyperplasia, and extent of fibrosis in the synovial tissues). To asses the degree of cartilage destruction a score from 0 to 4 was used (0 = no destruction, 1 = unequivocal erosions of less than 10% of cartilage and bone cross sections, 2 = erosion of 10–25%, 3 = erosion of 25–50%, 4 = erosion of more than 50% of cartilage and bone cross sections) [40, 42]. Additional histological sections were stained with safranin O to determine the loss of proteoglycan in the cartilage matrix, using scoring system as used for the evaluation of inflammatory features.

### Histomorphometric analysis of bone structure

Histomorphometric analysis of the bone structure was performend in the secondary spongiosa consisting of lamellar bone which represents homogeneous bone tissue. Secondary spongiosa is beginning at a distance of 1,25 mm from growth plate and was differentiated from primary spongiosa by means of morphological criteria (absence of cartilage cores) [43, 44].

In the secondary spongiosa trabecular bone volume and histomorphometric parameters of bone formation and bone resorption were evaluated by standard histomorphometry [41, 45, 46]. For tetracycline labeling, all animals received intraperitoneal injections of 45 mg/kg tetracycline (Supramycine; Grünenthal, Stolberg, Germany) at a volume of 1 ml PBS on day 22 and 25 after AIA induction.

Single-labeled and double-labeled surface as well as the mean distance between double labels were measured at uncoulored bone sclices of the same region used for histomorphometry by fluorescence microscopy (Axioplan, Carl Zeiss, Jena, Germany). Based on the measurements, parameters of bone formation and mineralization were calculated [47].

The measured parameters of standard histomorphometry of the trabecular bone of the secondary spongiosa are listed.

A) Bone volume
  1. Trabecular bone volume in relationship to tissue volume (%)
  2. Osteoid volume in relationship to bone volume (%).
B) Bone resorption

Resorption surface with osteoclasts in relation to whole bone surface (%) [i.e. levels of osteoclastic bone resorption].

C) Bone formation
  1. Osteoid-covered surface in relation to whole bone surface (%) [i.e. bone surface covered with non-mineralized, newly formed bone matrix].
  2. Osteoid-covered surface with osteoblasts in relation to whole bone surface (%) [i.e. levels of cellular bone formation].
  3. Mineralizing surface.
  4. Mineral apposition rate (MAR), calculated by mean distance between double labels divided by the interval labeling time (3 days) (μm/day)
  5. Bone formation rate (BFR/BS), calculated as MAR × MS/BS (μm3/μm2/day × 10-2).
  $$MS/\mathrm{OS}\;\left(\%\right)=\;\frac{\mathrm{single}‒\mathrm{labeled}\;\mathrm{surface}\;+\;\mathrm{double}‒\mathrm{labeled}\;\mathrm{surface}}{2}$$

### Statistical analysis

Data were presented as means ± standard deviation. The data were analysed statistically using the SPSS for Windows Statistical Programme [48]. Data were subjected to the non-parametric Kruskall-Wallis-analysis and, subsequently to the non-parametric Mann–Whitney U-test. Differences of p < 0.05 were considered significant.

## Results

### Influence of 1,25(OH)_2_D_3_ therapy on arthritis severity

**Figure 1:**
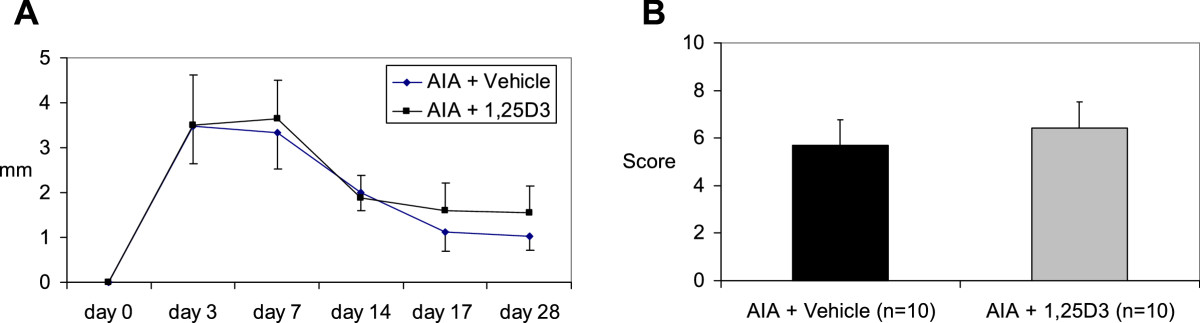
**Knee joint swelling (A) and histopathologic score (sum of the semiquantitative scores expressed as points for both, inflammation and joint destruction, see** **Methods** **for details) of arthritis on day 28 after AIA induction (B).** The knee joint swelling measured as difference between right and left joint during the time course of AIA as well as the histopathologic signs of inflammation and joint destruction were not influenced by 1,25(OH)_2_D_3_ therapy.

After arthritis induction rats developed rapid inflammation indicated by the acute joint swelling with a maximum between day 3 and 7 (Figure 1A). The clinical disease activity measured as joint swelling during the course of arthritis as well as the histological signs of inflammation and joint destruction as sum of the semiquantitative scores expressed as points for both, inflammation and joint destruction on day 28 after AIA induction were not influenced by 1,25(OH)_2_D_3_ treatment (Figure 1A and 1B).

### Influence of AIA on periarticular and axial bone (secondary spongiosa)

**Figure 2:**
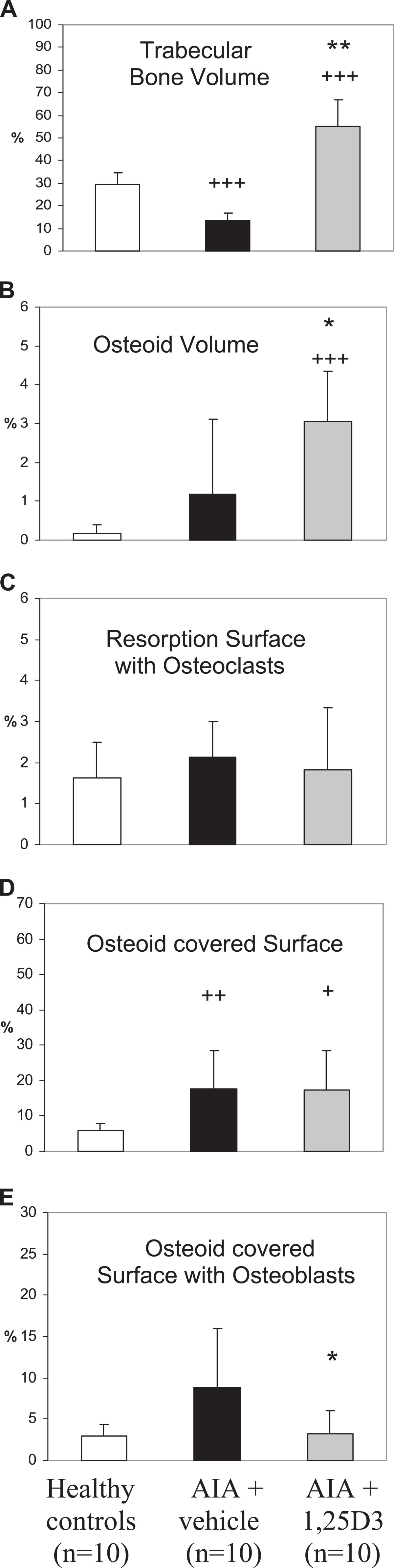
**Influence of 1,25(OH)**_**2**_ **D**_**3**_ **therapy on trabecular bone volume, osteoid volume and cellular bone turnover parameters (secondary spongiosa of the right tibia head – arthritic joint) in AIA of the rat.** In comparison to healthy controls, vehicle-treated AIA (AIA + vehicle) was associated with a highly significant decrease of trabecular bone volume **(A)**, a significant increase in osteoid-covered surface **(D)** and a numerical increase in osteoid volume **(B)** and osteoid-covered surface with osteoblasts **(E)**. 1,25(OH)_2_D_3_ therapy completely inhibited AIA-induced bone loss and resulted in a significant increase of trabecular bone volume **(A)** and osteoid volume **(B)** in comparison to vehicle-treated AIA and healthy controls. Osteoid-covered surface with osteoblasts was reduced by 1,25(OH)_2_D_3_ treatment to values of healthy controls **(E)**. Osteoid-covered surface remained increased in comparison to healthy animals **(D)**. Resorption surface with osteoclasts was not significantly influenced by AIA and by 1,25(OH)2D3 therapy **(C)**. **p < 0.01; *p < 0.05 vs. AIA + vehicle; +++p < 0.001; ++p < 0.01; +p < 0.05 vs. healthy controls.

**Figure 3:**
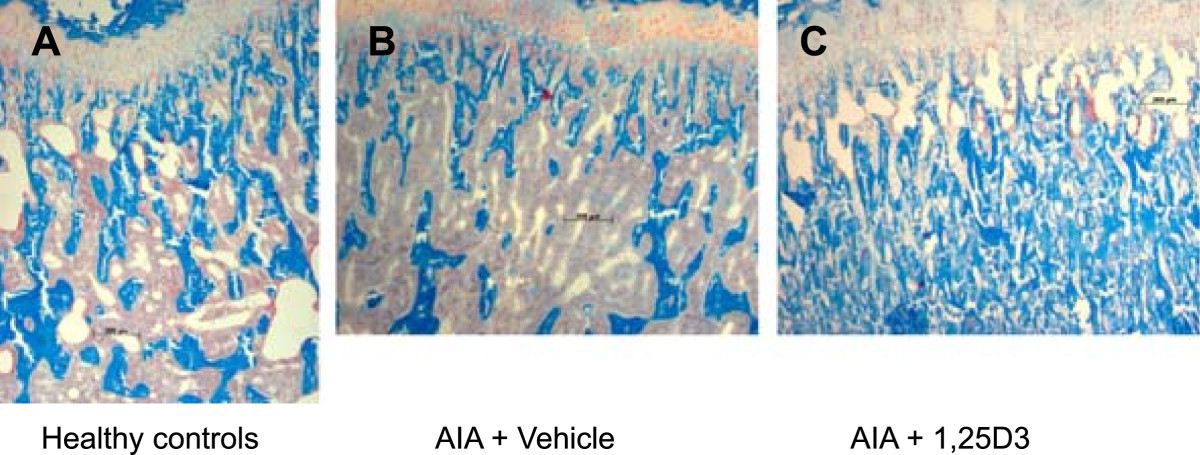
**Assessment of bone histology, secondary spongiosa of the right tibia head (representative Masson/Goldner stained sections).** In comparison to healthy controls **(A)**, vehicle-treated AIA resulted in a significant bone loss in secondary spongiosa **(B)**. Therapy with 0.2 μg/kg 1,25(OH)_2_D_3_ prevented AIA-induced bone loss completely and resulted in a highly significant increase in trabecular bone volume in comparison to both vehicle-treated AIA and healthy controls **(C)**.

**Figure 4:**
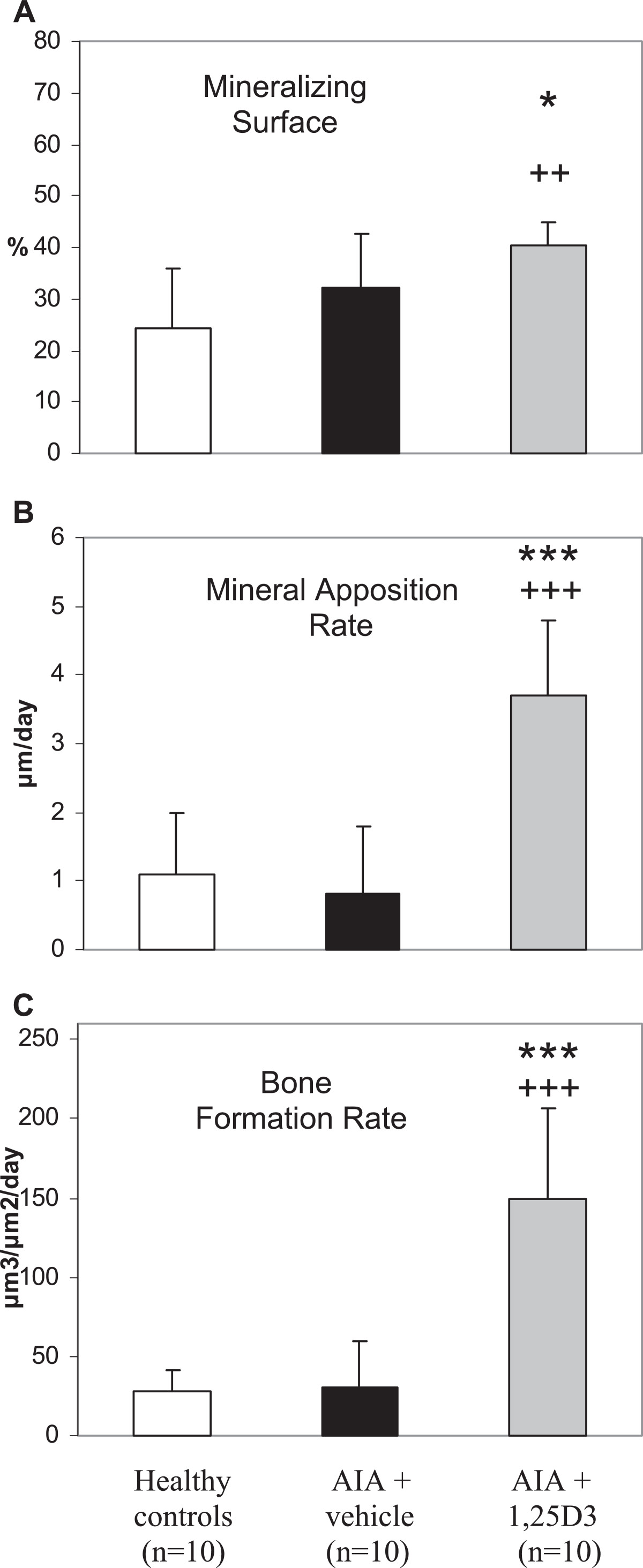
**Influence of 1,25(OH)**_**2**_ **D**_**3**_ **therapy on parameters of bone formation measured by tetracylin labeling (secondary spongiosa of the right tibia head – arthritic joint) in AIA of the rat.** Mineralizing surface **(A)**, mineral apposition rate **(B)** and bone formation rate **(C)** were unaffected by AIA. Treatment with 1,25(OH)_2_D_3_ resulted in a highly significant increase of mineral apposition rate and bone formation rate and a significant increase in mineralizing surface compared with vehicle-treated AIA and healthy controls. ***p < 0.001; *p < 0.05 vs. AIA + vehicle; +++p < 0.001; ++p < 0.01 vs. healthy controls.

**Figure 5:**
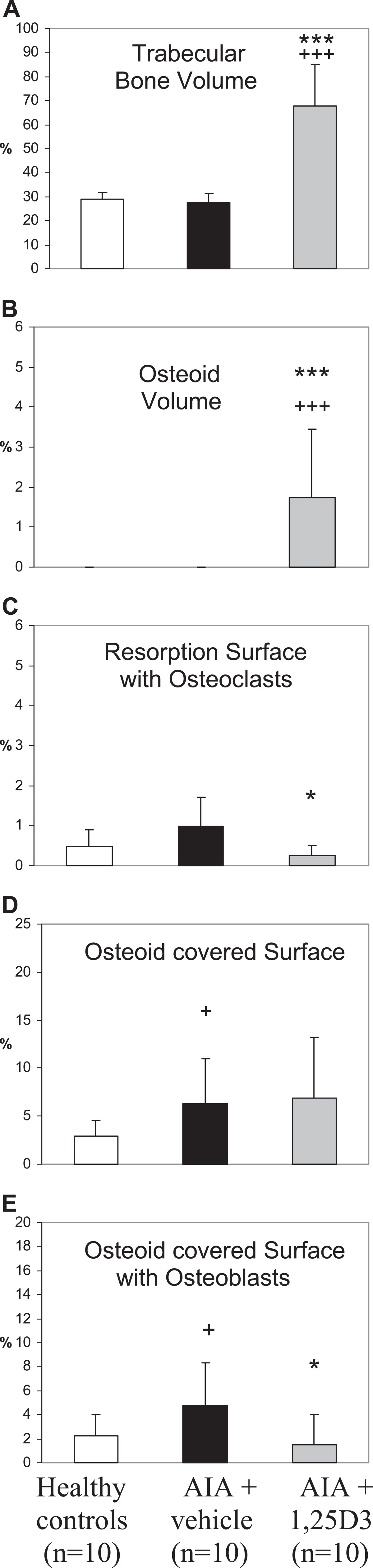
**Influence of 1,25(OH)**_**2**_ **D**_**3**_ **therapy on trabecular bone volume, osteoid volume and cellular bone turnover parameters (secondary spongiosa of the third lumbar vertebra) in AIA of the rat.** Vehicle-treated AIA was associated with a significant increase of osteoid-covered surface and osteoid-covered surface with osteoblasts **(D**; **E)** and a numerical increase in resorption surface with osteoclasts **(C)** in comparison with healthy controls. Trabecular bone volume and osteoid volume at the axial bone remained unaffected by AIA **(A**; **B)**. 1,25(OH)_2_D_3_ therapy led to a highly significant increase of trabecular bone volume **(A)** and osteoid volume **(B)** in comparison to vehicle-treated AIA and healthy controls and resulted furthermore in a significant decrease in resorption surface with osteoclasts **(C)** and osteoid-covered surface with osteoblasts **(E)** in comparison to vehicle-treated AIA and reduced these parameters to the values of healthy controls. ***p < 0.001; *p < 0.05 vs. AIA + vehicle; +++p < 0.001; +p < 0.05 vs. healthy controls.

**Figure 6:**
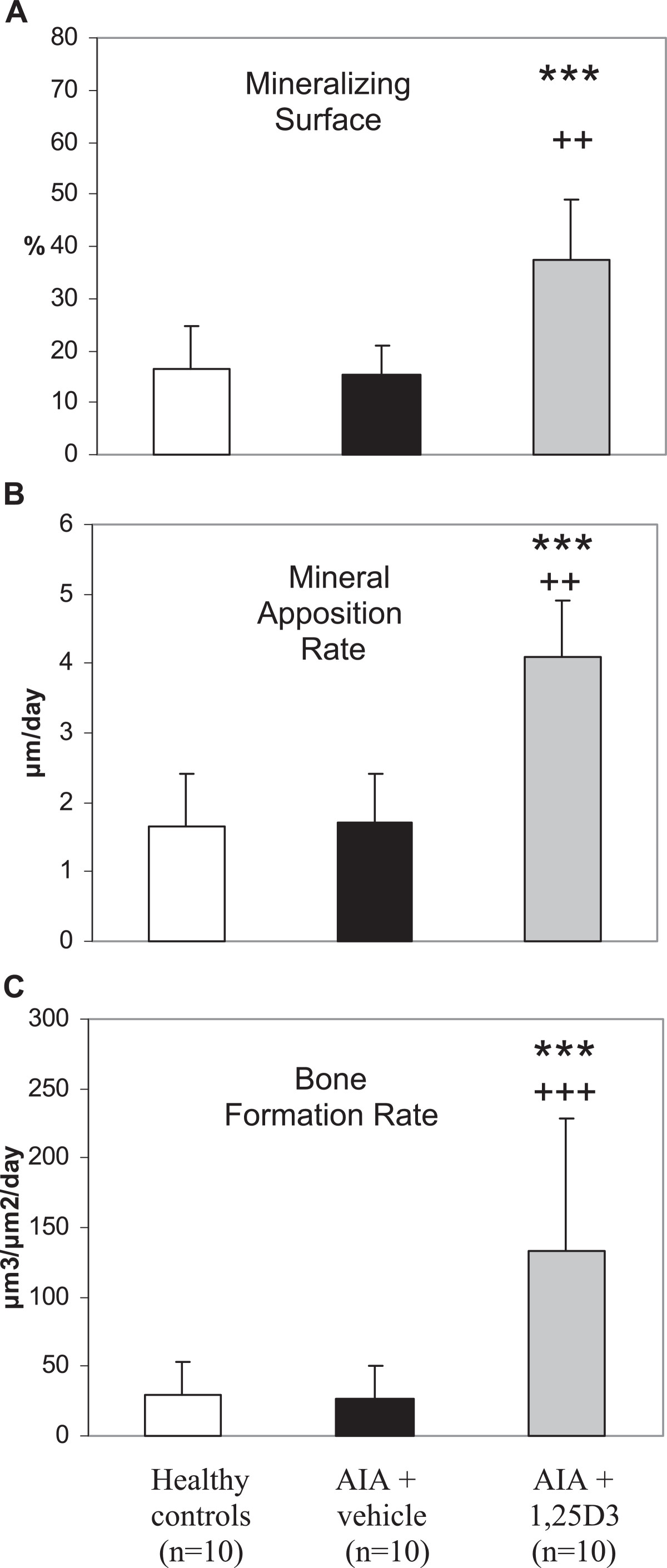
**Influence of 1,25(OH)**_**2**_ **D**_**3**_ **therapy on parameters of bone formation measured by tetracylin labeling (secondary spongiosa of the third lumbar vertebra) in AIA of the rat.** Mineralizing surface **(A)**, mineral apposition rate **(B)** and bone formation rate **(C)** were unaffected by AIA. 1,25(OH)_2_D_3_ therapy resulted in a highly significant increase of mineralizing surface, mineral apposition rate and bone formation rate in comparison to both vehicle-treated AIA and healthy controls. ***p < 0.001 vs. AIA + vehicle; +++p < 0.001; ++p < 0.01 vs. healthy controls.

AIA resulted in a highly significant decrease in trabecular bone volume of the secondary spongiosa of the right tibia head (periarticular bone of the arthritic joint; p < 0.001, Figures 2A and 3B). Arthritic animals were characterized by a numerical increase of resorption surface with osteoclasts (Figure 2C) and a significant increase of osteoid-covered surface and osteoid-covered surface with osteoblasts at periarticular bone (p < 0.01, Figure 2D,E). Mineralizing surface, mineral apposition rate and bone formation rate at the periarticular bone were not influenced by AIA (Figure 4A-C). In contrast, trabecular bone volume of the axial bone (third lumbar vertebra) was unaffected by AIA (Figure 5A). Resorption surface with osteoclasts was numerically increased, osteoid-covered surface and osteoid-covered surface with osteoblasts at the axial bone were significantly increased in arthritic animals (p < 0.05, Figure 5C,D,E). The parameters of bone formation measured by tetracycline labeling were not influenced by AIA at the axial bone (Figure 6A-C).

### Influence of treatment with 1,25(OH)_2_D_3_ on the secondary spongiosa of the right tibia head (periarticular bone)

1,25(OH)_2_D_3_ therapy led to a significant increase in trabecular bone volume (Figures 2A and 3C) not only in comparison to vehicle-treated AIA (p < 0.01) but also compared to healthy animals (p < 0.01). Furthermore, a significant increase in osteoid volume in comparison to vehicle-treated AIA (p < 0.05) and to healthy animals (p < 0.001) was observed with 1,25(OH)_2_D_3_ therapy (Figure 2B). Resorption surface with osteoclasts and osteoid-covered-surface were not influenced by 1,25(OH)_2_D_3_ as compared to vehicle-treated AIA (Figure 2C and 2D). Accordingly, osteoid-covered surface in animals receiving 1,25(OH)_2_D_3_ was significantly higher in comparison to healthy animals (p < 0.05). In contrast to osteoid-covered surface, osteoid-covered surface with osteoblasts was significantly reduced by 1,25(OH)_2_D_3_ therapy compared with vehicle-treated AIA (p < 0.05), resulting in a decrease of this parameter to the values of healthy animals (Figure 2E). Despite a decrease in osteoid-covered surface with osteoblasts, both mineralizing surface and mineral apposition rate were significantly increased by 1,25(OH)_2_D_3_ therapy in comparison to vehicle-treated AIA (p < 0.05 and p < 0.001, respectively) and compared to healthy animals (p < 0.01 and p < 0.001, respectively, Figure 4A and 4B). The increase of these both parameters of bone formation resulted in a highly significant increase of bone formation rate in 1,25(OH)_2_D_3_-treated rats in comparison to both vehicle-treated AIA and healthy animals (p < 0.001, Figure 4C).

### Influence of treatment with 1,25(OH)_2_D_3_ on the secondary spongiosa of the third lumbar vertebra (axial bone)

According to the findings at the periarticular bone, both trabecular bone volume and osteoid volume were highly significant increased by 1,25(OH)_2_D_3_ treatment in comparison to vehicle-treated AIA and healthy animals (p < 0.001, Figure 5A and 5B). Both resorption surface with osteoclasts and osteoid-covered surface with osteoblasts were significantly reduced by 1,25(OH)_2_D_3_ therapy in comparison to vehicle-treated AIA (p < 0.05, Figure 5C and 5E) to levels according to those of healthy animals. Osteoid-covered surface remained numerically higher in comparison to healthy animals in 1,25(OH)_2_D_3_-treated rats (Figure 5D). Mineralizing surface and mineral apposition rate were significantly increased during 1,25(OH)_2_D_3_ therapy in comparison to vehicle-treated AIA (p < 0.001) and compared to healthy animals (p < 0.01; Figure 6A and 6B). Accordingly, 1,25(OH)_2_D_3_ treatment resulted in a highly significant increase in bone formation rate in comparison to both vehicle-treated AIA and healthy animals (p < 0.001, Figure 6C).

## Discussion

A suppressive effect of 1,25(OH)_2_D_3_ therapy on inflammation and joint destruction was not observed in our study. From the background of the T cell-dependence of AIA, this is surprising, because important immunomodulatory effects of 1,25(OH)_2_D_3_ are mediated by the modulation of T cell differentiation contributing to protective effects of 1,25(OH)_2_D_3_ in different T cell-mediated diseases [16–19, 23]. The most probable explanation for the missing anti-inflammatory effect of 1,25(OH)_2_D_3_ in the present study may be the relatively short 1,25(OH)_2_D_3_ administration period in the prearthritic phase of AIA beginning only on day 3 before arthritis induction. In contrast to our investigation, in studies with evidence for inhibitory effects of active vitamin D metabolites on incidence, severity and/or progression of arthritis in collagen-induced arthritis (CIA) of rats [30, 33] and mice [32] and in adjuvant arthritis of the rat [29] treatment was initiated prior to immunization or at the time point of immunization or the animals were treated over a longer period during the prearthritic phase, respectively. Due to the long time period between the two immunizations on day 21 an day 14 before arthritis induction and the first administration of 1,25(OH)_2_D_3_ in our study, it is probable that a substantial part of T cells has been differentiated into the proinflammatory Th17 and Th1 cells before 1,25(OH)_2_D_3_ application.

In contrast to the missing anti-inflammatory effect, we observed substantial effects of 1,25(OH)_2_D_3_ therapy on periarticular and axial bone.

The main finding of our study is the dramatic osteoanabolic effect of 1,25(OH)_2_D_3_ therapy resulting in an important increase in trabecular bone volume in comparison to untreated AIA rats and healthy controls at both periarticular and axial bone, despite the ineffectiveness of the treatment on the inflammation. Interestingly, the increase in parameters of bone formation and mineralization such as bone formation rate in 1,25(OH)_2_D_3_-treated AIA rats occurs despite a significant reduction of osteoid-covered surface with osteoblasts in comparison to untreated AIA indicating an increase of the capacity of the single osteoblast to form new bone due to treatment with active vitamin D hormone. Although the direct effects of 1,25(OH)_2_D_3_ on osteoblasts are dependent on the species examined, the time course of 1,25(OH)_2_D_3_ treatment and the differentiation state of osteoblasts, an osteoanabolic vitamin D receptor-mediated signaling in mature osteoblasts has been clearly shown in other studies [14, 15, 49]. Osteoanabolic effects of 1,25(OH)_2_D_3_ include an increase of the expression of genes involved in mineralization such as alkaline phosphatase and osteocalcin [13, 14, 50, 51]. Although the findings on the effect of 1,25(OH)_2_D_3_ on mineralization are not consistent and dose-dependent [52], a stimulation of mineralization by higher doses of 1,25(OH)_2_D_3_ has been demonstrated in vitro [53] and in vivo in rats at doses of 0.125 and 0.2 μg/kg/day [54, 55]. In a recent study it has been shown, that suppression of interferon-ß mediated inhibitory effects on mineralization by 1,25(OH)_2_D_3_ may be critical for the stimulatory effects of 1,25(OH)_2_D_3_ on mineralization [56].

Interestingly, the resorption surface with osteoclasts was not increased by 1,25(OH)_2_D_3_ at the periarticular bone in our study and even significantly reduced at the axial bone. This result is surprising, because one of the most evident actions of 1,25(OH)_2_D_3_ on the bone is the induction of RANKL in osteoblasts resulting in a stimulation of osteoclastogenesis [35]. An indirect effect of 1,25(OH)_2_D_3_ on bone resorption mediated by reduction of inflammation resulting in a secondary decrease of bone resorption can be excluded as a cause for this observation, because 1,25(OH)_2_D_3_ has clearly no suppressive effect on inflammation in our treatment protocol. Despite the induction of RANKL, 1,25(OH)_2_D_3_-mediated inhibitory mechanisms on bone resorption have also been described. Thus, 1,25(OH)_2_D_3_ has been shown to interfere with RANK-mediated signaling by inhibiting the induction of c-Fos in a dose-dependent manner via vitamin D receptor (VDR) in osteoclast precursor cells resulting in an inhibition of their differentiation into mature osteoclasts [57]. In addition, an inhibitory effect of 1,25(OH)_2_D_3_ on the differentiation of osteoclast precursors associated with a decreased RANK expression and an increased expression of the CCAAT enhancer-binding protein, an inhibitor of osteoclastogenesis on these cells has been proved in osteoclast precursors of normal peripheral blood and of synovial fluid of RA patients [58]. Furthermore, the RANKL/OPG ratio which is critical for the regulation of bone resorption is dependent on the differentiation state of the osteoblast [10, 59]. For both human and mouse osteoblastic cells a decrease in RANKL/OPG ratio and in their osteoclastogenic potential, respectively, has been shown during the differentiation into mature osteoblasts [59–64]. The capacity of mineralisation is a feature of the mature osteoblast. Therefore, the finding of our study of a combination of an increase in mineralization with a reduction of osteoid-covered surface with osteoclasts is in accordance with the assumption, that 1,25(OH)_2_D_3_ treatment may induce the differentiation process of osteoblasts resulting in both increase in mineralization and a decrease in the osteoclastogenic potential of osteoblasts. Furthermore, a suppression of PTH secretion by 1,25(OH)_2_D_3_ or by 1,25(OH)_2_D_3_-induced slight hypercalcemia may also contribute to the reduction of resorption surface with osteoclasts.

Because of the finding of reduced 1,25(OH)_2_D_3_ levels in both adjuvant arthritis of the rat [27, 28] and in RA patients with high disease activity [24] and with respect to the insignificant increase in periarticular osteoid volume in untreated AIA as a potential feature for slight osteomalacia, the question arises, if 1,25(OH)_2_D_3_ administration and effects in our study reflects in part a correction of an inflammation-associated 1,25(OH)_2_D_3_ deficiency. Although, serum levels of vitamin D metabolites were not measured in this study, this assumption is not probable, because 1,25(OH)_2_D_3_ serum levels in untreated AIA (25.5 ± 16.6 pg/ml, n = 7) have been found to be not reduced compared to healthy animals (15.8 ± 15.6 pg/ml; n = 8; n.s.) in an earlier investigation [65].

## Conclusions

In summary, the results of our study indicate that the administration of active vitamin D hormone completely inhibited arthritis-induced bone loss by stimulation of mineralization at periarticular and axial bone and by inhibition of bone resorption at the axial bone despite missing effects on inflammation. The main cause of this beneficial effect of 1,25(OH)_2_D_3_ may be the induction of osteoblast differentiation resulting in both increasing mineralizing capacity of the single osteoblast and reduced osteoclastogenic potential of the mature osteoblast. Both an adequate vitamin D supply and the administration of active vitamin D metabolites could counteract and inhibit important mechanisms of bone loss in rheumatoid arthritis and should be used as an adjuvant therapeutic principle in this disease.

## Authors’ original submitted files for images

Below are the links to the authors’ original submitted files for images. Authors’ original file for figure 1 Authors’ original file for figure 2 Authors’ original file for figure 3 Authors’ original file for figure 4 Authors’ original file for figure 5 Authors’ original file for figure 6 Authors’ original file for figure 7

## Abbreviations

1: 25(OH)_2_D_3_: 1,25-dihydroxyvitamin D3
AIA: Antigen-induced arthritis
CIA: Collagen-induced arthritis
mBSA: Methylated bovine serum albumin
OPG: Osteoprotegerin
PBS: Phosphate-buffered saline
RA: Rheumatoid arthritis
RANKL: Receptor of NFkappaB ligand
VDR: Vitamin D receptor.
