# Supplementary figures and images for: 1,25-Dihydroxyvitamin D3 prevents bone loss of the secondary spongiosa in arthritic rats by an increase of bone formation and mineralization and inhibition of bone resorption
Source: BMC Musculoskelet Disord. 2014 Oct 14;15:345. doi: 10.1186/1471-2474-15-345 (PMC4210592; doi:10.1186/1471-2474-15-345)

**A**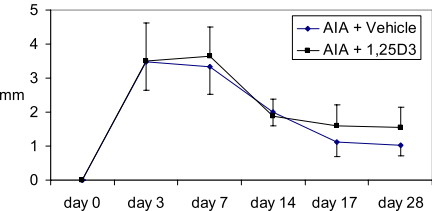**B**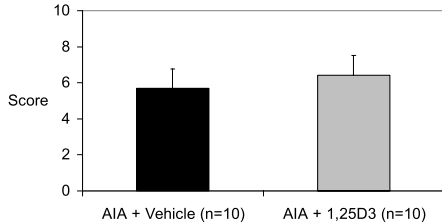

Supplement: Supplementary file 1 — Authors’ original file for figure 1 [file 12891_2014_2283_MOESM1_ESM.pdf]

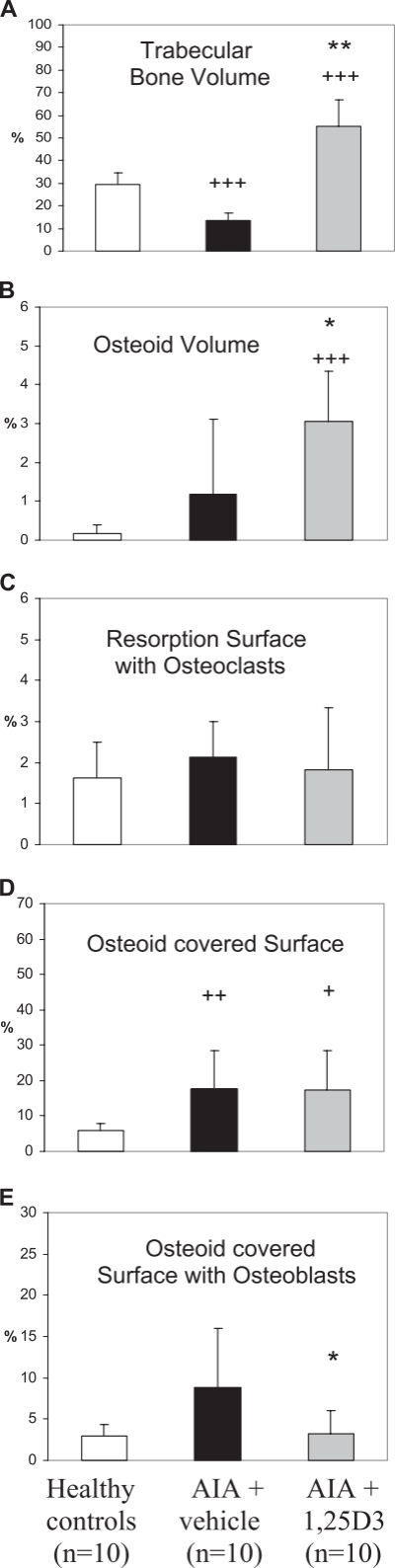

Supplement: Supplementary file 2 — Authors’ original file for figure 2 [file 12891_2014_2283_MOESM2_ESM.pdf]

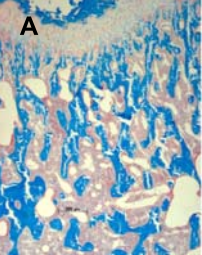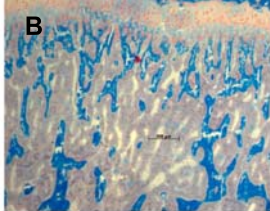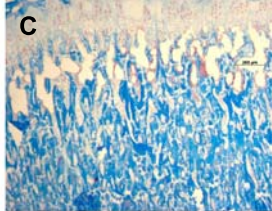

Healthy controls

AIA + Vehicle

AIA + 1,25D3

Supplement: Supplementary file 3 — Authors’ original file for figure 3 [file 12891_2014_2283_MOESM3_ESM.pdf]

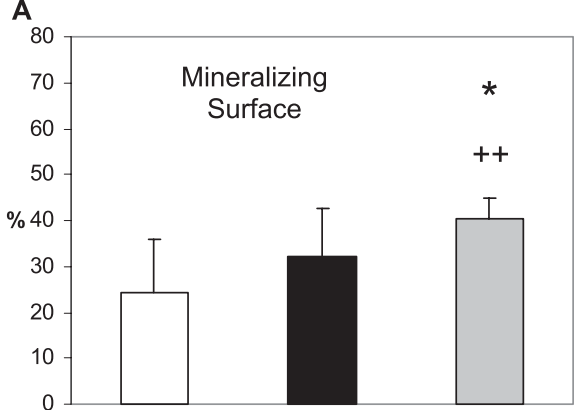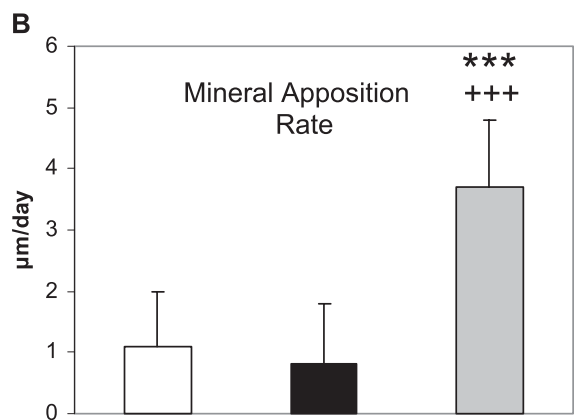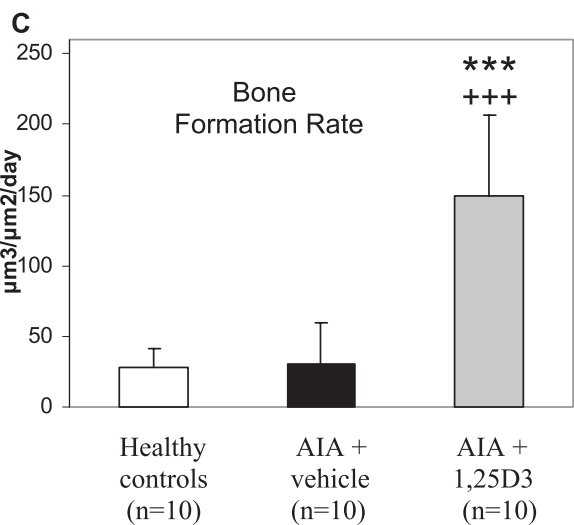

Supplement: Supplementary file 4 — Authors’ original file for figure 4 [file 12891_2014_2283_MOESM4_ESM.pdf]

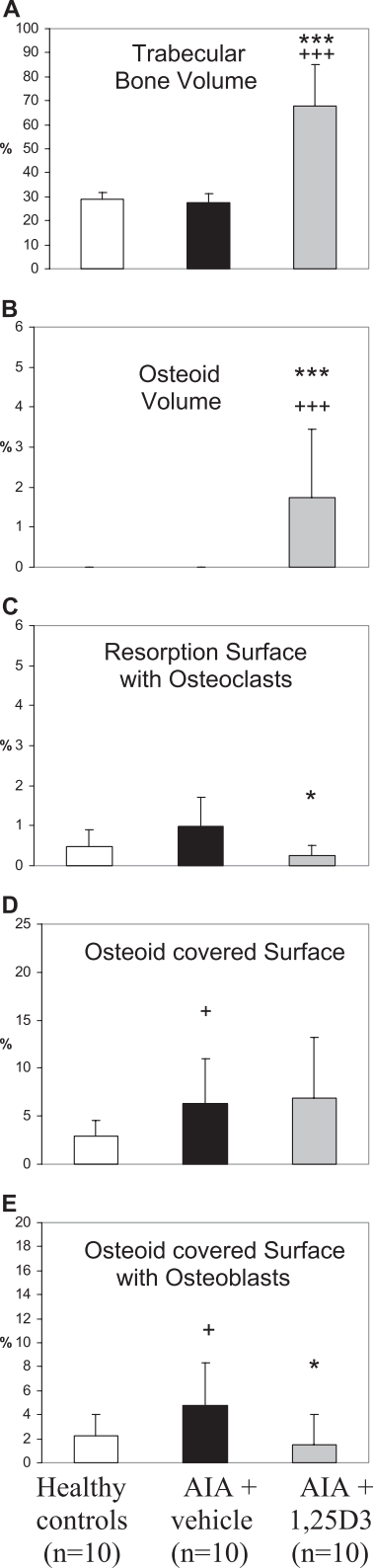

Supplement: Supplementary file 5 — Authors’ original file for figure 5 [file 12891_2014_2283_MOESM5_ESM.pdf]

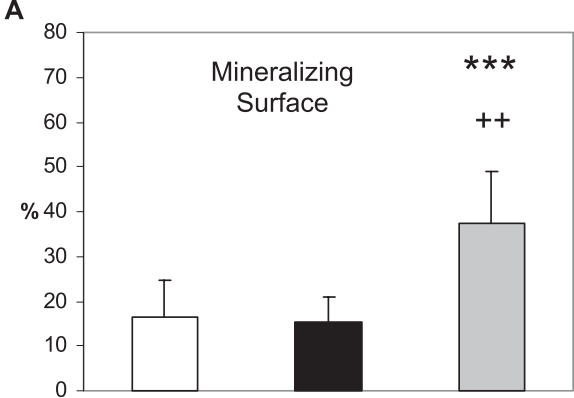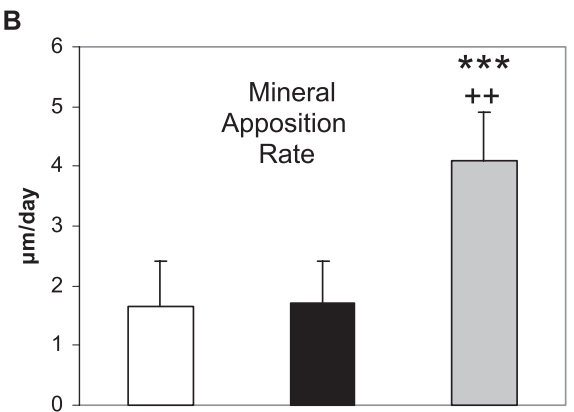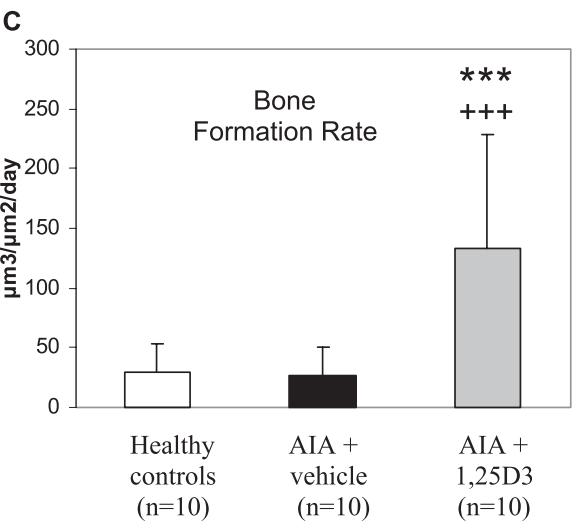

Supplement: Supplementary file 6 — Authors’ original file for figure 6 [file 12891_2014_2283_MOESM6_ESM.pdf]
