# Supplementary material for: 1,25-Dihydroxyvitamin D3 prevents bone loss of the secondary spongiosa in arthritic rats by an increase of bone formation and mineralization and inhibition of bone resorption
Source: BMC Musculoskelet Disord. 2014 Oct 14;15:345. doi: 10.1186/1471-2474-15-345 (PMC4210592; doi:10.1186/1471-2474-15-345)
Supplement: Supplementary file 7 — Authors’ original file for figure 7 [file 12891_2014_2283_MOESM7_ESM.doc]

**Table 1**: Parameters of standard histomorphometry of trabecular bone

1. Bone volume
   1. Trabecular bone volume in relationship to tissue volume (%)
   2. Osteoid volume in relationship to bone volume (%)
2. Bone resorption

Resorption surface with osteoclasts in relation to whole bone surface (%) [i.e. levels of osteoclastic bone resorption]

1. Bone formation
   1. Osteoid-covered surface in relation to whole bone surface (%) [i.e. bone surface covered with non-mineralized, newly formed bone matrix]
   2. Osteoid-covered surface with osteoblasts in relation to whole bone surface (%) [i.e. levels of cellular bone formation]
   3. Mineralizing surface

MS/OS (%) = single-labeled surface + double-labeled surface

2

4) Mineral apposition rate (MAR), calculated by mean distance between double

labels divided by the interval labeling time (3 days) (µm/day)

5) Bone formation rate (BFR/BS),

calculated as MAR x MS/BS (µm^3^/µm^2^/day x 10^-2^)
